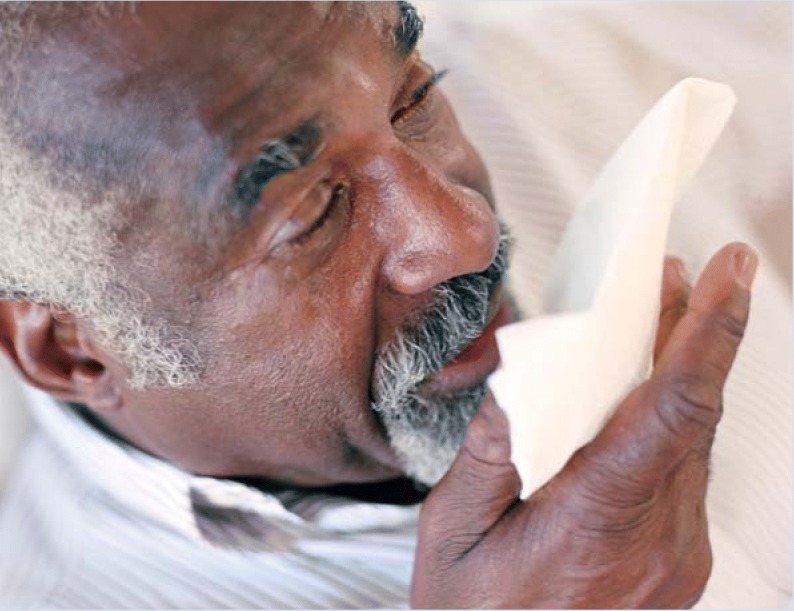# Headliners: Immune Response: Lead Disrupts T Cell Function

**Published:** 2006-01

**Authors:** Tanya Tillett

Farrer DG, Hueber SM, McCabe MJ Jr. 2005. Lead enhances CD4^+^ T cell proliferation indirectly by targeting antigen presenting cells and modulating antigen-specific interactions. Toxicol Appl Pharmacol 207:125–137.

Although lead has been banned from use in products like house paint, gasoline, and water pipe solder in the United States, it is still present in older housing, and is used in products in other countries. Besides its widely studied neurotoxicity, lead is also a well-known immunotoxicant, though little is known about its mechanism of action. Now NIEHS grantee Michael McCabe and colleagues at the University of Rochester have discovered how lead may work to disturb T cell function in the body.

Previous studies have suggested that lead’s immunotoxic effects may occur at exposures even lower than those required for neurotoxicity to occur; thus, suboptimal immune function may affect people who do not even realize they have been exposed to lead. Older adults and lactating, pregnant, and postmenopausal women are at greater risk for lead exposure as lead stored in the bones is released back into the blood and soft tissues. Children are also at heightened risk for lead exposure because they engage in more hand-to-mouth activity and absorb a larger proportion of ingested lead across the intestinal epithelium than do adults.

The Rochester researchers used flow cytometry to analyze T cell division in cell cultures derived from lead-treated mice. T cells help regulate the body’s immune system by attacking bacteria, viruses, foreign tissue, and tumor cells. At day 4 of treatment, the frequency of proliferating T cells was much greater in treated than in nontreated cultures. Lead appeared to target a type of cell known as antigen presenting cells, and its effect was based on specific peptide-major histocompatibility complex conjugate. The results suggest that lead may pose even more long-term health threats than originally thought.

## Figures and Tables

**Figure:**